# Clinical characteristics of patients with unexplainable hypothalamic disorder diagnosed by the corticotropin-releasing hormone challenge test: a retrospective study

**DOI:** 10.1186/s12902-022-01237-7

**Published:** 2022-12-09

**Authors:** Yuji Hataya, Marie Okubo, Takuro Hakata, Kanta Fujimoto, Toshio Iwakura, Naoki Matsuoka

**Affiliations:** 1 Department of Diabetes and Endocrinology, Kobe City Medical Center General Hospital, 2-1-1, Minatojima Minamimachi, Chuo-ku, Kobe, Hyogo 650-0047 Japan; 2 Department of Diabetes, Endocrinology and Nutrition, Kyoto University Graduate School of Medicine, 54 Kawaharacho, Shogoin, Sakyo-ku Kyoto, 606-8507 Japan

**Keywords:** Central adrenal insufficiency, Hypocortisolemia, Corticotropin-releasing hormone challenge test, Insulin tolerance test, Myalgic encephalitis/chronic fatigue syndrome

## Abstract

**Background:**

The corticotropin-releasing hormone (CRH) challenge test can distinguish the disorders of the hypothalamus from those of the pituitary. However, the pathophysiology of hypothalamic disorder (HD) has not been fully understood. This study aimed to elucidate the clinical characteristics of patients with unexplainable HD, diagnosed by the CRH challenge test.

**Methods:**

We retrospectively reviewed patients who underwent the CRH challenge test. Patients were categorized into four groups as follows: patients with peak serum cortisol ≥18 μg/dL were assigned to the normal response (NR) group (*n* = 18), among patients with peak serum cortisol < 18 μg/dL and peak adrenocorticotropic hormone (ACTH) increase ≥two-fold, patients without obvious background pathology were assigned to the unexplainable-HD group (*n* = 18), whereas patients with obvious background pathology were assigned to the explainable-HD group (*n* = 38), and patients with peak serum cortisol < 18 μg/dL and peak ACTH increase <two-fold were assigned to the pituitary disorder (PD) group (*n* = 15). Inter-group comparisons were performed based on clinical characteristics.

**Results:**

In the CRH challenge test, the peak plasma ACTH levels were significantly lower in the unexplainable-HD group than in the NR group, despite more than two-fold increase compared to basal levels. The increase in serum cortisol was significantly higher in the unexplainable-HD group than in the explainable-HD and PD groups. Although patients in the unexplainable-HD group showed a clear ACTH response in the insulin tolerance test, some patients had peak serum cortisol levels of < 18 μg/dL. Furthermore, attenuated diurnal variations and low normal levels of urinary free cortisol were observed. Most patients in the unexplainable-HD group were young women with chronic fatigue. However, supplementation with oral hydrocortisone at physiological doses reduced fatigue only in some patients.

**Conclusions:**

Patients with unexplainable HD diagnosed by the CRH challenge test had hypothalamic–pituitary–adrenal (HPA) axis dysfunction and some patients had mild central adrenal insufficiency. Hydrocortisone supplementation reduced fatigue only in some patients, suggesting that HPA axis dysfunction may be a physiological adaptation. Further investigation of these patients may help elucidate the pathophysiology of myalgic encephalitis/chronic fatigue syndrome.

## Background

Central adrenal insufficiency (AI) is caused by disorders of the hypothalamus and pituitary [1]. The common cause of central AI is discontinuation of exogenous glucocorticoid use and the presence of tumors and inflammatory diseases. In unexplainable central AI, patients with no other pituitary hormone deficits are diagnosed with idiopathic isolated adrenocorticotropic hormone (ACTH) deficiency [2]. Most cases of idiopathic isolated ACTH deficiency are presumably caused by the destruction of corticotroph cells in the pituitary due to an autoimmune mechanism. Some cases have been reported to be caused by hypothalamic disorder (HD); however, the pathophysiology of HD is poorly understood [2].

The usefulness of several dynamic tests for assessing the hypothalamic–pituitary–adrenal (HPA) axis has been reported. The insulin tolerance test (ITT) is considered the gold standard for diagnosing central AI [1]. Insulin-induced hypoglycemia is considered to act on the hypothalamus, where it strongly stimulates ACTH secretion by inducing the release of both corticotropin-releasing hormone (CRH) and arginine vasopressin (AVP). However, owing to the risk of hypoglycemia, ITT is contraindicated in individuals with ischemic heart disease, cerebrovascular disease, or seizures. Recently, the high-dose (250 μg) short synacthen test (SST) and low-dose (1 μg) SST have been widely used for diagnosing central AI [3]. The rationale for using SST to diagnosis central AI is the assumption that acute responsiveness of the adrenal zona fasciculata is attenuated in chronic endogenous ACTH deficiency. The CRH challenge test is not widely used because of its low sensitivity for diagnosing central AI [4]. However, it is known that this challenge test can distinguish the disorders of the hypothalamus from those of the pituitary by directly stimulating the pituitary [2]. Specifically, patients without a decreased ACTH response despite a decreased cortisol response are considered to have HD. However, the diagnostic criterion for HD is not well defined and, to our knowledge, there were no studies that have investigated these patients. Therefore, this study aimed to elucidate the clinical characteristics of patients with unexplainable HD diagnosed using the CRH challenge test.

## Materials and methods

### Participants and procedure

We retrospectively reviewed the medical records of 89 consecutive patients, aged ≥17 years, who underwent the CRH challenge test to assess pituitary function (excluding patients with iatrogenic and endogenous Cushing’s syndrome), at Kobe City Medical Center General Hospital, Japan, between August 2016 and April 2021. The definition of response to the CRH challenge test was based on the guidelines of the Japan Endocrine Society [5]. Patients with peak serum cortisol levels ≥18 μg/dL in the CRH challenge test were assigned to the normal response (NR) group. Among patients with peak serum cortisol levels < 18 μg/dL and peak ACTH increase ≥two-fold compared to basal ACTH levels, patients without obvious background pathology were assigned to the unexplainable-HD group, whereas patients with obvious background pathology were assigned to the explainable-HD group. Patients with peak serum cortisol levels < 18 μg/dL and peak ACTH increase <two-fold compared to basal ACTH levels were assigned to the pituitary disorder (PD) group. The basal, peak, and increased levels of plasma ACTH and serum cortisol in the CRH challenge test were compared among the four groups. Furthermore, the clinical characteristics and clinical course of the unexplainable-HD group were examined.

Patients included in this study undertook the challenge tests (CRH and ITT) in accordance with standard procedures on separate consecutive days. Blood samples for the evaluation of circadian rhythms of ACTH and cortisol were collected at 0900, 1600, and 2200 h. Urinary free cortisol (UFC) was measured using 24-h urine collection. Patients receiving oral hydrocortisone as a replacement therapy were administered the last dose at least 24 h before the study. Magnetic resonance imaging or computed tomography was performed in all patients to investigate the lesions that could cause central AI.

### Challenge tests for ACTH and cortisol secretion

The test commenced at 0900 h after overnight fasting (12 h). The protocols for each test differed as follows: (1) CRH challenge test - a single 100-μg human CRH dose was injected intravenously, and blood samples were collected before injection and 30, 60, 90, and 120 min after injection; and (2) ITT - regular insulin (at a dose of 0.1 U/kg body weight) was injected intravenously, and blood samples were collected before injection and 30, 60, 90, and 120 min after injection. Plasma glucose levels < 40 mg/dl after insulin injection were a prerequisite for interpreting the results.

### Assays for ACTH, cortisol, and UFC

The collected blood samples were separated and stored until ACTH and cortisol assays were conducted. Plasma ACTH and serum cortisol levels were measured using an electrochemiluminescence immunoassay (ECLusys ACTH and cortisol II kit, Roche Diagnostics, Tokyo, Japan), with intra- and inter-assay coefficients of variation (CVs) of < 3.5 and < 3.7%, respectively. The standard institutional reference ranges for morning plasma ACTH and serum cortisol were 7.2–63.3 pg/mL and 7.1–19.6 μg/dL, respectively. UFC was measured using a radioimmunoassay (Cortisol kit FR, Fujirebio, Tokyo, Japan), with intra- and inter-assay CVs of < 8.6 and < 7.6%, respectively. The standard reference range for UFC was 11.2–80.3 μg/24 h.

### Ethics approval and consent to participate

This study was approved by the Research Ethics Committee of Kobe City Medical Center General Hospital (approval no. zn210812) and conducted in accordance with the principles of the Declaration of Helsinki. The requirement for informed consent was waived by the Research Ethics Committee of Kobe City Medical Center General Hospital due to the retrospective nature of this study.

### Statistical analyses

The normality of distribution of the continuous variables was assessed using the Kolmogorov–Smirnov test. Continuous data are expressed as median with interquartile range (IQR), and categorical data are expressed as numbers (percentages), unless stated otherwise. Continuous data were compared across multiple groups using the Kruskal–Wallis test, and the Steel–Dwass method was used for multiple comparisons. Categorical data were compared using the chi-square test, corrected by Bonferroni’s method for multiple comparisons. Statistical significance was set at *P* < 0.05. Analyses were performed using the Statistical Package for the Social Sciences version 27.0 (IBM SPSS 27.0; Armonk, NY: IBM Corp) and JMP 16 (SAS Institute Inc., Cary, NC).

## Results

The clinical characteristics of each group are shown in Table 1. The median (IQR) age of the patients in the unexplainable-HD group was 39.0 (32.5–46.5) years, which was significantly lower than that of patients in the explainable-HD and PD groups. The prevalence of women in the unexplainable-HD group was 77.8%, which was significantly higher than that in the explainable-HD and PD groups. The median (IQR) body mass index (BMI) of patients in the unexplainable-HD group was 19.6 kg/m^2^ (18.9–22.6 kg/m^2^), which was significantly lower than that of patients in the explainable-HD group. The prevalence of patients receiving hydrocortisone replacement therapy at the time of examination in the unexplainable-HD group was 33.3%; this was significantly lower than that in the explainable-HD and PD groups. However, age, sex, BMI, and number of patients receiving hydrocortisone replacement did not differ between the unexplainable-HD and NR groups. The unexplainable-HD group was not associated with any other pituitary hormone deficiency. No association with autoimmune diseases, including the presence of thyroid autoantibodies, was found. However, the prevalence of psychiatric disorders was higher in the unexplainable-HD group than in the explainable-HD group.

**Table 1:** Comparison of clinical characteristics of the study groups

	NR *n* = 18	unexplainable-HD *n* = 18	explainable-HD *n* = 38	PD *n* = 15
Age, y	58.5 (36.5–69.8)	39.0 (32.5–46.5)	58.0 (47.5–72.8) ^d^	59.0 (49.0–67.5) ^c^
Women, n (%)	14 (77.8)	14 (77.8)	15 (39.5) ^a, c^	3 (20.0) ^b, d^
Body mass index, kg/m^2^	23.3 (21.5–25.7)	19.6 (18.9–22.6)	24.2 (21.8–26.8) ^c^	21.2 (20.6–22.9)
Hydrocortisone replacement, n (%)	10 (55.6)	6 (33.3)	27 (71.1) ^c^	11 (73.3) ^c^
Hypothalamic and pituitary lesions, n (%)				
Tumoral (post-surgery)	9 (50.0)	0 (0) ^b^	20 (52.6) ^d^	4 (26.7)
Tumoral (non-surgery)	4 (22.2)	0 (0)	11 (29.0)	0 (0)
irAE	0 (0)	0 (0)	2 (5.3)	6 (40.0) ^b, d^
Empty sella syndrome	0 (0)	0 (0)	2 (5.3)	1 (6.7)
Sheehan syndrome	0	0	1 (2.6)	0
Sarcoidosis	0	0	1 (2.6)	0
Pituitary stalk transection	0	0	1 (2.6)	0
Hypophysitis	2 (11.1)	0 (0)	0 (0)	0 (0)
Unknown	3 (16.7)	18 (100) ^b^	0 (0) ^d^	4 (26.7) ^d^
Other pituitary hormone deficiencies, n (%)	6 (33.3)	0 (0)	26 (68.4) ^d^	4 (26.7)
Growth hormone	4	0	22	3
Prolactin	1	0	9	2
Thyrotropin	5	0	16	3
Gonadotropin	3	0	22	2
Antidiuretic hormone	2	0	3	0
Comorbidities, n (%)				
Autoimmune disease	1 (5.6)	3 (16.7)	5 (13.2)	1 (6.7)
Thyroid autoantibody	2/4 (50.0)	3/10 (30.0)	4/11 (36.4)	1/9 (11.1)
Allergic disease	0 (0)	6 (33.3) ^b^	3 (7.9) ^c^	1 (6.7)
Psychiatric disorder	1 (5.6)	5 (27.8)	0 (0) ^d^	1 (6.7)
Depression	0	4	0	0
ADHD	0	1	0	0
Schizophrenia	0	1	0	0
Panic disorder	0	0	0	1
Anxiety disorder	1	0	0	0

Continuous data were compared by the Kruskal–Wallis test and the Steel–Dwass method for multiple comparisons. Categorical data were compared using the chi-square test corrected by Bonferroni’s method. Data are expressed as median (interquartile range) and n (%). ^a^*P* < 0.05 and ^b^*P* < 0.01 compared with the NR group, and ^c^*P* < 0.05 and ^d^*P* < 0.01 compared with the unexplainable-HD group. *ADHD *attention deficit hyperactivity disorder, *explainable-HD *explainable-hypothalamic disorder group, *irAE *immune-related adverse event, *n *number of patients, *NR *normal response group, *PD *pituitary disorder group, *unexplainable-HD *unexplainable-hypothalamic disorder group

**Fig. 1:**
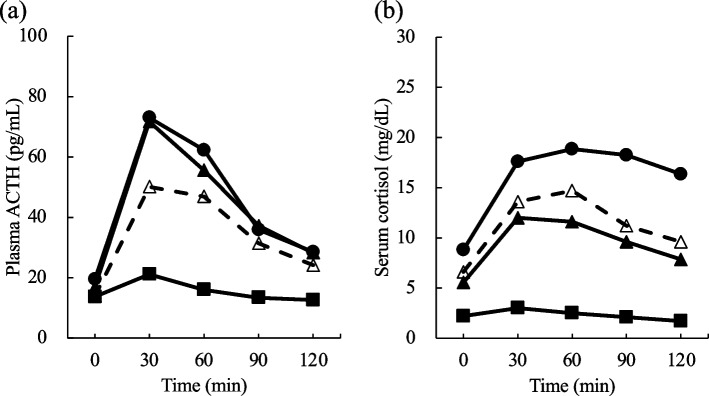
Time course of plasma ACTH (**a**) and serum cortisol (**b**) levels following CRH stimulation. The normal response (closed circle), unexplainable-hypothalamic disorder (open triangle), explainable-hypothalamic disorder (closed triangle), and pituitary disorder (closed square) groups after 100-μg CRH injection over time. Data are presented as median values. ACTH, adrenocorticotropic hormone; CRH, corticotropin-releasing hormone

**Fig. 2:**
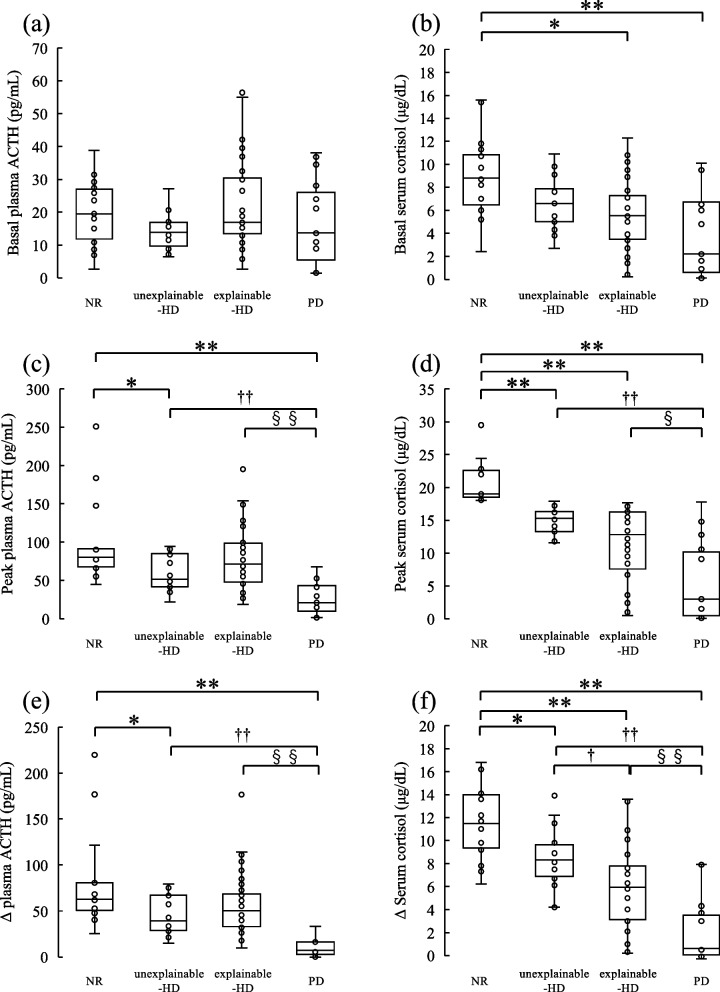
Box-whisker plot showing ACTH and cortisol levels. (**a**) Basal plasma ACTH, (**b**) basal serum cortisol, (**c**) peak plasma ACTH, (**d**) peak serum cortisol, (**e**) increase in plasma ACTH, and (**f**) increase in serum cortisol. The Kruskal–Wallis test was used to compare the study groups, and the Steel–Dwass method was used for multiple comparisons. *P < 0.05 and **P < 0.01 compared with the NR group, ^†^*P* < 0.05 and ^††^*P* < 0.01 compared with the unexplainable-HD group, and ^§^*P* < 0.05 and ^§§^*P* < 0.01 compared with the explainable-HD group. ACTH, adrenocorticotropic hormone; explainable-HD, explainable-hypothalamic disorder group; NR, normal response group; PD, pituitary disorder group; unexplainable-HD, unexplainable-hypothalamic disorder group

The time course of plasma ACTH and serum cortisol levels following CRH stimulation are shown in Fig. 1. The peak ACTH response was 30 min in all groups. Conversely, the peak cortisol response was 60 min in the unexplainable-HD group and the NR group, but 30 min in the explainable-HD group and the PD group. Median values and the box plots for basal levels, peak levels, and increments are shown in Table 2 and Fig. 2. The basal plasma ACTH levels were similar among the four groups. The basal serum cortisol level in the unexplainable-HD group tended to be lower than that in the NR group and higher than that in the PD group. The peak level and increase in plasma ACTH were significantly lower in the unexplainable-HD group than in the NR group, despite more than two-fold increase compared to basal levels. The unexplainable-HD and explainable-HD groups did not differ in the peak level and increase in plasma ACTH. The peak level and increase in serum cortisol were significantly higher in the unexplainable-HD group than in the PD group. The peak serum cortisol level in the unexplainable-HD group tended to be higher than that in the explainable-HD group, but there was no significant difference. However, the increase in serum cortisol was significantly higher in the unexplainable-HD group than in the explainable-HD group.

**Table 2:** Corticotropin-releasing hormone challenge test

	NR	unexplainable-HD	explainable-HD	PD
Basal plasma ACTH (pg/mL)	19.5 (11.9–27.0)	13.9 (9.6–16.9)	16.9 (13.5–30.4)	13.7 (5.5–26.1)
Basal serum cortisol (μg/dL)	8.8 (6.5–10.9)	6.6 (5.0–7.9)	5.6 (3.5–7.3) ^a^	2.2 (0.6–6.7) ^b^
Peak plasma ACTH (pg/mL)	80.3 (68.0–91.2)	51.5 (41.9–84.8) ^a^	71.7 (48.1–98.7)	21.1 (9.8–43.2) ^b, d, f^
Peak serum cortisol (μg/dL)	19.1 (18.6–22.6)	15.3 (13.3–16.4) ^b^	12.8 (7.6–16.3) ^b^	3.0 (0.5–10.2) ^b, d, e^
Δ Plasma ACTH (pg/mL)	62.7 (50.6–80.8)	39.2 (28.8–67.2) ^a^	50.4 (32.9–68.4)	7.0 (2.8–16.4) ^b, d, f^
Δ Serum cortisol (μg/dL)	11.5 (9.4–14.0)	8.3 (6.9–9.7) ^a^	6.0 (3.1–7.8) ^b, c^	0.6 (0.1–3.5) ^b, d, f^

Data are expressed as median (interquartile range). The Kruskal–Wallis test was used to compare the study groups, and the Steel–Dwass method was used for multiple comparisons. ^a^*P* < 0.05 and ^b^*P* < 0.01 compared with the NR group, ^c^*P* < 0.05 and ^d^*P* < 0.01 compared with the unexplainable-HD group, and ^e^*P* < 0.05 and ^f^*P* < 0.01 compared with the explainable-HD group. *ACTH* adrenocorticotropic hormone, *explainable-HD* explainable-hypothalamic disorder group, *NR* normal response group, *PD* pituitary disorder group, *unexplainable-HD* unexplainable-hypothalamic disorder group

The subjective symptoms of each group are shown in Table 3. The most common symptom of patients in the unexplainable-HD group was chronic fatigue, which was significantly more common than in the NR and explainable-HD groups. The number of patients with a depressive state and insomnia in the unexplainable-HD group were higher than those of other groups.

**Table 3:** Comparison of symptoms between groups

	NR *n* = 18	unexplainable-HD *n* = 18	explainable-HD *n* = 38	PD *n* = 15
Chronic fatigue	4 (22.2)	18 (100.0) ^b^	8 (21.1) ^d^	11 (73.3) ^b^
Depressive state	0 (0)	5 (27.8) ^b^	0 (0) ^d^	0 (0) ^d^
Insomnia	0 (0)	4 (22.2) ^a^	0 (0) ^d^	1 (6.7)
Appetite loss	1 (5.6)	0 (0)	6 (15.8)	3 (20.0)
Nausea	0 (0)	1 (5.6)	3 (7.9)	4 (26.7) ^a^
Weight loss	2 (11.1)	4 (22.2)	1 (2.6)	2 (13.3)
Slight fever	0 (0)	2 (11.1)	2 (5.3)	0 (0)
Arthritis/myalgia	0 (0)	2 (11.1)	2 (5.3)	0 (0)
Visual disturbance	4 (22.2)	0 (0)	12 (31.6) ^c^	1 (6.7)
Headache	1 (5.6)	2 (11.1)	4 (10.5)	4 (26.7)
Dizziness	0 (0)	2 (11.1)	2 (5.3)	0 (0)
Asymptomatic	7 (38.9)	0 (0) ^a^	10 (26.3)	0 (0) ^a^

Categorical data were compared using the chi-square test corrected by Bonferroni’s method for multiple comparisons. Data are expressed as n (%). ^a^*P* < 0.05 and ^b^*P* < 0.01 compared with the NR group, and ^c^*P* < 0.05 and ^d^*P* < 0.01 compared with the unexplainable-HD group. *Explainable-HD* explainable-hypothalamic disorder group, *n *number of patients, *NR* normal response group, *PD* pituitary disorder group, *unexplainable-HD *unexplainable-hypothalamic disorder group

**Fig. 3:**
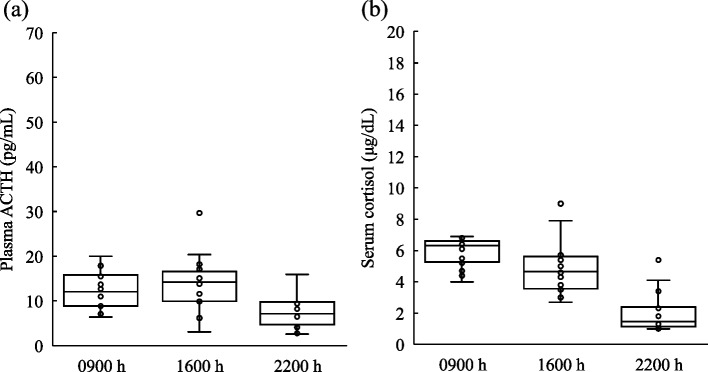
Box-whisker plot showing circadian rhythms of ACTH and cortisol. Circadian rhythm of plasma ACTH (**a**) and serum cortisol (**b**) levels of patients in the unexplainable-hypothalamic disorder group (*n* = 16) at 0900, 1600, and 2200 h. ACTH, adrenocorticotropic hormone

**Fig. 4:**
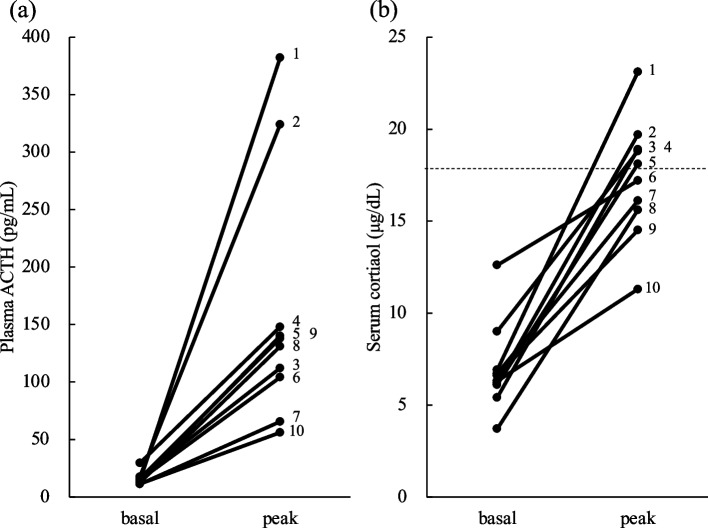
Insulin tolerance test in the patients with unexplainable-hypothalamic disorder without contraindications (*n* = 10). The basal and peak levels of plasma ACTH (**a**) and serum cortisol (**b**). ACTH, adrenocorticotropic hormone

The circadian rhythms of ACTH and cortisol were evaluated in 16 patients in the unexplainable-HD group (Fig. 3). Although serum cortisol levels were low throughout the day, the circadian rhythms were preserved. The median (IQR) UFC levels in the 24-h urine collection were a low normal (38.3 [32.0–50.8] μg/24 hr). ITT was performed in 10 patients of the unexplainable-HD group who had no contraindications and agreed to the challenge test (Fig. 4). All patients had decreased plasma blood glucose levels (< 40 mg/dl), and the peak plasma ACTH levels increased to at least twice the basal plasma ACTH levels (range, 56.1 to 382.0 pg/mL); peak serum cortisol levels ranged from 11.3 to 23.1 μg/dL, and were < 18 μg/dL in five patients.

Five patients with peak serum cortisol levels < 18 μg/dL in the ITT and four patients who did not undergo ITT were treated with oral hydrocortisone at physiologic doses (5–10 mg/day). Two of the former five and the latter four showed improvement in symptoms. Furthermore, we followed up these six patients (follow-up period: 9–40 months). Four patients with persistently low levels of random serum cortisol (range, 4.6 to 7.0 μg/dL) continued hydrocortisone therapy. Three patients with normalized random serum cortisol levels (range, 9.2 to 10.2 μg/dL) discontinued hydrocortisone and had no symptom subsequently.

## Discussion

We investigated patients categorized into four according to their response to CRH stimulation. Patients in the PD group showed a markedly decreased ACTH and cortisol response. It is presumed that damage to pituitary ACTH-secreting cells themselves leads to decreased ACTH secretion. Patients in the explainable-HD group showed a decreased cortisol response and a similar ACTH response to that of the NR group. It is presumed that in most cases, mechanical compression by tumoral lesions causes an inadequate secretion of endogenous ACTH due to impaired transport of CRH and AVP from the hypothalamus to the pituitary, followed by attenuated adrenal responsiveness [6]. Large variations in ACTH response in the explainable-HD group may be associated with complications of damage to the ACTH-secreting cells themselves, or with various conditions depending on the degree and duration of the disorder. Patients in the unexplainable-HD group showed not only a decreased cortisol response but also an attenuated ACTH response compared with those of the NR group. These differences in response between the unexplainable-HD and NR groups suggest that the patients in the unexplainable-HD group have HPA axis dysfunction. The higher cortisol responses of the unexplainable-HD group compared with those of the explainable-HD and PD groups indicate that the attenuated adrenal response was mild. However, some patients showed peak serum cortisol levels of < 18 μg/dL in the ITT, suggesting that they have mild central AI.

The HPA axis is controlled by neuroendocrine neurons in the paraventricular nucleus (PVN) of the hypothalamus [7]. When these neurons are stimulated, CRH and AVP are released and transported to the anterior pituitary gland. Although AVP does not drive ACTH release on its own, it can synergize with CRH to greatly enhance ACTH release. The PVN receives circadian inputs from the suprachiasmatic nucleus and stress inputs from the brainstem and limbic areas. Stress can be divided into physical stress and psychological stress [8]. Information on physical stress is communicated directly to the PVN from sensory organs. In contrast, information on psychological stress is transmitted from multiple limbic regions to the PVN via excitatory and inhibitory neurons. The preservation of circadian rhythms and the clear response of ACTH to hypoglycemia in the unexplainable-HD group suggested that the pathways from the suprachiasmatic nucleus and physical stress were not impaired. Nevertheless, patients in the unexplainable-HD group showed attenuated ACTH response to CRH stimulation compared with those in the NR group. One of the hypotheses that can explain the mechanism of this abnormal response is the dysregulation of the PVN [8]. Specifically, the PVN is assumed to be over suppressed, followed by a decrease of CRH and AVP secretion. Therefore, ACTH may have not be synergistically released by exogenous CRH. The attenuated diurnal variation and low normal levels of UFC in the unexplainable-HD group can be explainable by the suppressed state of PVN. The CRH challenge test is not widely available because of the large variability in cortisol responses in normal subjects, as well as low sensitivity and specificity for diagnosing central AI [4]. However, using the CRH challenge test to identify HD may be useful for elucidating its pathophysiology.

Most patients in the unexplainable-HD group had undergone the CRH challenge test to examine hypocortisolemia, detected during the screening for chronic fatigue. When patients present with persistent (≥6 months) and unexplained fatigue, it is necessary to consider myalgic encephalomyelitis/chronic fatigue syndrome (ME/CFS) [9]. There are many reports on the association between ME/CFS and HPA axis dysfunction. Patients with ME/CFS reportedly have normal circadian rhythms but attenuated diurnal variation [10, 11]. Conflicting results have been reported for ACTH responses to the CRH challenge tests between patients with ME/CFS and controls [12–15]. However, Scott et al. showed that ACTH response to CRH stimulation was decreased in patients with ME/CFS, and coadministration of desmopressin (an AVP analog) with CRH normalized the blunted ACTH response [16]. This result suggests that a decrease in endogenous AVP could contribute to the attenuated ACTH response to CRH stimulation. Most studies that evaluated patients with ME/CFS using ITT showed no significant differences in ACTH response between patients with ME/CFS and controls [14, 17, 18]. The patient population in this retrospective study could not be confirmed to be consistent with the diagnostic criteria for ME/CFS. However, similarities in endocrinological findings between patients with unexplainable HD and patients with ME/CFS suggest that they may have similar pathophysiology.

Chronic stress is known to activate the HPA axis in different ways, including chronic basal hypersecretion, sensitized stress responses, and adrenal exhaustion [19]. Furthermore, in individuals at high risk of fatigue, HPA axis changes have been reported to develop as a consequence of chronic fatigue [20]. These changes are considered a physiological adaptive response to chronic stress but may become pathological depending on the circumstances of the stressor [19]. Randomized controlled trials have demonstrated the efficacy of hydrocortisone treatment in reducing fatigue only in some patients with ME/CFS [21, 22]. These results suggest that HPA axis dysfunction in patients with ME/CFS may be a physiological adaptation. We administered oral hydrocortisone at physiological doses to nine patients in the unexplainable-HD group: among them, subjective symptoms improved in six patients. However, this retrospective analysis was not controlled with a comparative analysis, and the subjective improvements were not quantified using a validated questionnaire. Therefore, it is unclear whether the administration of hydrocortisone was effective. The patients with unexplainable HD had more severe fatigue despite a slight attenuation of the cortisol level, and hydrocortisone treatment reduced fatigue only in some patients. These findings suggest that HPA axis dysfunction in the patients with unexplainable HD may also be a physiological adaptation. Due to the side effects of hydrocortisone use and the risk of suppression of the endogenous HPA axis, the distinction between unexplainable HD and other central AIs is clinically important.

It has been reported that the autoimmune process may underlie the onset of hypothalamic AI [23–25], but autoimmune involvement was not observed in this study population. However, we did not investigate anti-hypothalamic and anti-pituitary antibodies in the present study. The pathogenesis of unexplainable HD may be heterogeneous, underscoring the need for further studies to elucidate the pathogenesis of HD. The unexplainable-HD group had a high proportion of women. This is similar to the sex differences observed in ME/CFS [26], which may be associated with the pathophysiology of the syndrome. Many patients in the unexplainable-HD group experienced depression. Depression can be divided into the following two subtypes: melancholic depression with relative overactivity on the HPA axis and atypical depression with relative hypoactivity in the HPA axis [27]. HPA axis abnormality in patients with depression may also have a similar pathophysiology.

This study has some limitations. First, we categorized each group using only the response in the CRH challenge test. Therefore, these groups may not accurately reflect the sites of the disorder. Furthermore, we did not include the SST results in the analysis of this study, because it was performed only in some patients. However, borderline AI is difficult to diagnose rigorously even with SST and must be diagnosed with additional challenge tests and patient backgrounds [3]. The cut-off levels of response to the challenge test were determined according to the criteria of the guidelines [5]. However, the cut-off level for cortisol response may vary among assays, and ideally, criteria for an abnormal response should be validated locally [3]. In future research, it will be necessary to determine appropriate examinations and cut-off levels and clarify the diagnostic criteria for HD. Second, as this was a retrospective study, we could not perform comparisons with healthy controls. All patients without lesions who complain of fatigue may be better interpreted by classifying them according to their results in the CRH challenge test and comparing them directly with healthy controls. Third, confounding factors that affect the HPA axis, such as the frequent use of herbal and other complementary medicines, physical activity, sleep disorders, and working night shifts, could not be excluded. Fourth, some patients were taking hydrocortisone as replacement therapy, which may have affected their subjective symptoms. However, they stopped taking it during the examination of the HPA axis, so as not to affect the results. Fifth, this study did not consider the effects of corticosteroid-binding globulin (CBG). It has been reported that CBG may be affected by chronic stress and plays a role in the adjustment of the HPA axis [19]. Further studies are needed to measure the CBG concentration and free cortisol concentration.

## Conclusions

Patients with unexplainable HD diagnosed by the CRH challenge test had HPA axis dysfunction, while some patients had mild central AI. Oral hydrocortisone at physiological doses reduced fatigue only in some patients, suggesting that HPA axis dysfunction may be a physiological adaptation. Further investigation of these patients may help elucidate the pathophysiology of ME/CFS.

## Data Availability

The datasets analyzed during the current study are available from the corresponding author on reasonable request.

## Abbreviations

ACTH: adrenocorticotropic hormone
AI: adrenal insufficiency
AVP: arginine vasopressin
BMI: body mass index
CBG: corticosteroid-binding globulin
CRH: corticotropin-releasing hormone
CV: coefficients of variation
HD: hypothalamic disorder
HPA: hypothalamic–pituitary–adrenal
IQR: interquartile range
ITT: insulin tolerance test
ME/CSF: myalgic encephalomyelitis/chronic fatigue syndrome
NR: normal response
PD: pituitary disorder
PVN: paraventricular nucleus
SST: short synacthen test
UFC: urinary free cortisol
